# Sensing Behavior of Metal-Free Porphyrin and Zinc Phthalocyanine Thin Film towards Xylene-Styrene and HCl Vapors in Planar Optical Waveguide

**DOI:** 10.3390/nano11071634

**Published:** 2021-06-22

**Authors:** Nuerguli Kari, Marco Zannotti, Rita Giovannetti, Patigu Maimaiti, Patima Nizamidin, Shawket Abliz, Abliz Yimit

**Affiliations:** 1 Institute of Applied Chemistry, College of Chemistry, Xinjiang University, Urumqi 830046, China; nurri7695@163.com (N.K.); 18195918820@stu.xju.edu.cn (P.M.); patiman207@sina.com (P.N.); shawket_abliz@sina.com (S.A.); 2 School of Science and Technology, Chemistry Division, University of Camerino, 62032 Camerino, Italy

**Keywords:** metal-free porphyrin, zinc phthalocyanine, spin-coated thin film, planar optical waveguide sensor, HCl vapor, xylene-styrene vapors

## Abstract

The sensing behavior of a thin film composed of metal-free 5, 10, 15, 20-tetrakis (p-hydroxy phenyl) porphyrin and zinc phthalocyanine complex towards m-xylene, styrene, and HCl vapors in a homemade planar optical waveguide (POWG), was studied at room temperature. The thin film was deposited on the surface of potassium ion-exchanged glass substrate, using vacuum spin-coating method, and a semiconductor laser light (532 nm) as the guiding light. Opto-chemical changes of the film exposing with hydrochloric gas, m-xylene, and styrene vapor, were analyzed firstly with UV-Vis spectroscopy. The fabricated POWG shows good correlation between gas exposure response and absorbance change within the gas concentration range 10–1500 ppm. The limit of detection calculated from the logarithmic calibration curve was proved to be 11.47, 21.08, and 14.07 ppm, for HCl gas, m-xylene, and styrene vapors, respectively. It is interesting to find that the film can be recovered to the initial state with trimethylamine vapors after m-xylene, styrene exposures as well as HCl exposure. The gas-film interaction mechanism was discussed considering protonation and π-π stacking with planar aromatic analyte molecules.

## 1. Introduction

Aromatic organic solvents such as m-xylene, styrene are common chemicals in almost every chemistry laboratory, while exposure to these solvents or their vapors causes health issues such as insomnia, damage to the neurological system, respiratory system, liver, and kidney. Instrumental methods such as high-performance liquid chromatography (HPLC) and high-performance capillary electrophoresis (HPCE), quantum resistive sensors (QRS) are generally used for vapor detection [1,2]. However, methods with low-cost and facile processing are needed to study and advance this field. Different materials are reported in the literature for the detection of aromatic compounds and HCl, for example, V_2_O_5_ doped ZnFe_2_O_4_ composited thin film was used to detect xylene and styrene at room temperature using optical waveguide [3], pentacene-based organic field-effect transistor (FET) was applied to detect HCl [4], terbium-based metal-organic frameworks (MOF) to detect styrene through fluorescence mechanism [5]. In addition, composite sensors of poly(styrene-acrylic acid) with TiO_2_ nanoparticles [6] and MOF/polymer-based photonic crystal [7] were applied for the detection of volatile aromatic hydrocarbon vapors.

In the detection of molecular species, optical sensing methods are very attractive. Planar Optical Waveguide (POWG) sensor represents an interesting system that consists of a waveguide layer, that usually is a potassium-ion-exchange glass substrate, and a sensor layer; the response of this sensor depends on the evanescent wave principle of laser light [8,9] The detection of the molecule is based on two important factors: the absorbance of the film, that is affected by the interaction with the analyte molecules and the change of the reflected light intensity from the POWG thin film that is related to the absorbance changes [10]. The analyte detection by POWG sensors has several advantages with respect to other sensor materials: high potential sensitivity, fast response and recovery times, ability to work at room temperature, anti-electromagnetic interference, remote monitoring, safe detection, simple structure with easy manufacturing, and particularly important, low production costs [11,12].

In this contest, optical waveguides composed of metal-oxides, organic dyes, or carbon nanomaterials are promising methods for gas exposure detection due to the optical and chemical changes within the sensing layer [9]. Hence, thin films deposited on a transparent glass substrate with large refractive index difference and low loss, display intense responses toward surface condition transformations [13]. 

Nonlinear optical organic compounds such as porphyrins (P) and phthalocyanines (Pc) are promising chemicals in optical waveguides to limit the laser radiation due to their large nonlinearities, inherently fast response, broadband spectral response, and ease of processing [14]. Ps are composed of four pyrrole subunits interconnected at α carbon position via methine bridges, while Pc has nitrogen instead of CH at the meso-position of the porphyrin, and the pyrroles are conjugate with four benzene rings. Therefore, porphyrins and phthalocyanines exhibit outstanding chemical and optical character due to their macrocyclic structure with 18 delocalized π electrons. Ps exhibit large excited-state absorption cross-sections, and long triplet excited state lifetimes, which enables porphyrins to serve as an effective optical limiter [14]; Pcs are similar to P and are also attracting extensive attention thanks to their nonlinear optical characteristics, strong light absorption properties in the visible region, and small dielectric constants [15]. Ps show a strong Soret band between 400–500 nm and less intense Q bands in the range between 550–650 nm; Pcs usually show a deep-blue color with a strong Q-band in the visible region and a weak Soret band in the UV-spectral region [16,17].

Because of the inner center of the macrocycle and high electron density, these dyes can coordinate with almost all metals and numerous volatile organic compounds through strong chemical bonds and weak non-covalent bonds such as hydrogen bonding, π-π interactions, or Van der Waals forces.

Due to their high molar absorption coefficient, high refractive index, and absorption/emission properties in violet and visible region, P and Pc are applied as solar cells [16], opto-chemical and electrochemical sensors [17]. The tendency of Pc to self-assemble on a substrate is higher than that of porphyrin, and Pc has been widely applied as light-harvesting/donor molecules in solar cells owing to high absorption extinction coefficients in the visible region, and p-type semiconducting behavior [18]. Refractive index (n) and extinction coefficient (k) of Pc film at visible spectra region were rather steady compared to ultra-violet region with a value as n = 1.6–1.9, k = 5 × 10^8^ M^−1^ cm^−1^ with optical energy gap equals to 1.97 eV [19].

P and Pc derivatives have been studied in the thin-film state for different optical chemical detection with various instrumental methods. In the application of ZnPc films, visible light absorption [20], the transition temperature of crystalline form, surface morphology, and the molecular alignment of Pc films grown on different substrates [21,22,23], were studied. Besides, P and metallo-Pc derivatives were fabricated on different substrates and used as opto-chemical detectors of HCl [17], alcohol vapors [24], amines, ketones, alkanes, and pyridine vapors [25]. All of these researches indicate that thin-film fabricated with the blend of derivatives of P and Pc can be considered as one of the promising candidates for gas sensors. 

In this work a new method cooperating UV-Vis spectrophotometer with homemade optical waveguide gas sensor, based on meso-tetra(p-hydroxyphenyl)porphyrin and Zn-phthalocyanine dyes deposited on potassium ion-exchanged glass substrate was reported.. In this application, a UV-Vis spectrophotometer was used to investigate the sensing behavior of HCl gas, m-xylene, and styrene vapor at room temperature. In fact, due to their π-extended aromatic structure, the investigated porphyrin shows a strong absorption band in the visible region at around 400 nm while Zn-phthalocyanine complex shows strong absorption at a longer wavelength at 600–700 nm; thus, the combination of these two compounds provides a photo-response that spans the entire visible spectrum by permitting to explore the absorption behavior of the film interacting with various amounts of vapors within 300–800 nm range at room temperature. 

## 2. Materials and Methods

### 2.1. Materials

5, 10, 15, 20-tetrakis (p-hydroxyphenyl) porphyrin (THPP), with 95% purity, was purchased from Frontier Scientific Inc.; zinc phthalocyanine (ZnPc) (95%, Tokyo Chemical Industry Co., Ltd., Tokyo, Japan), dimethylformamide (DMF), Trimethylamine (TMA), m-xylene, styrene, diiodomethane and hydrochloric acid (HCl) were purchased from Tianjin Zhiyuan Chemical Industry. All reagents were in analytical grade and used without pretreatment. 

### 2.2. Sensing Film Fabrication

THPP porphyrin (3.6 × 10^−6^ mol L^−1^) and ZnPc complex (2.02 × 10^−5^ mol L^−1^) were prepared in 2 mL of DMF, sonicated for 20 min, and used to cover a surface area (5 mm × 26 mm) at the center of a potassium ion-exchanged glass slide (76 mm × 26 mm × 1 mm, Matsunami Glass Ind., Ltd., Tokyo, Japan). The film was prepared using a vacuum spin-coater (KW-4A, Shanghai Kaimeite Artificial Technology) with the first rotating velocity as 500 rpm (5 s) and the second rotating velocity as 1400 rpm (25 s). 

**Figure 1:**
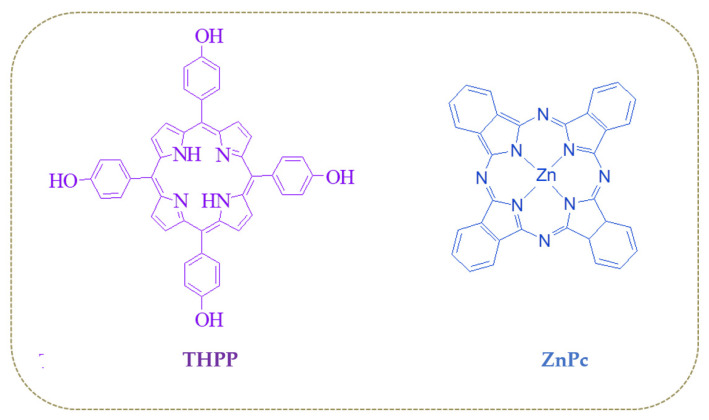
Chemical structures of the sensing film compositions: THPP porphyrin and ZnPc phthalocyanine.

Molecular structures of THPP porphyrin and ZnPc phthalocyanine are reported in Figure 1. 

THPP and ZnPc solutions were analyzed by UV-Vis 1780 spectrophotometer (SHIMADZU, Kyoto, Japan). In addition, THPP, ZnPc powders and the blend film deposited on the glass were characterized by X-ray diffraction (XRD, Kyoto, Japan DPMax 2400) equipped with graphite-monochromatized CuKα radiation (λ = 1.5418 Å), angle: 10–80°, and X-ray Photoelectron Spectroscopy (XPS) with chromatic Al Kα source (12.5 mA, 12 kV) at pressure as 2 × 10^−10^ Torr, respectively. The deconvolution of the XPS spectra was operated by Fityk software (Microsoft, GitHub, San Francisco, CA, USA). 

The films on potassium ion-exchanged glass substrate, before and after gas exposure, were analyzed by Scanning Electronic Microscope (SU8010, Hitachi, Tokyo, Japan); in this case, the samples were sputtered with gold to make the samples conductive and allow the acquisition of the images. 

### 2.3. Gas Preparation

Analyte vapors were prepared by static headspace sampling method; in this case, an aliquot of pure analyte was placed in the bottle (600 mL) and then, after 20 min at 60 °C, the proper volume of headspace gas from the bottle was taken with a micro-syringe. The higher concentrations of gases were prepared by vaporizing different volumes of liquid samples, while the lower concentrations of gases were prepared by diluting high-concentration-gas samples with dry air as a diluent.

The gas concentrations were calculated by the following Equation (1) [26]:

(1) $$c=\left(\frac{22.4\rho TV_{S}}{273MV}\right)\times 10^{3}$$

where *c* and *V* is the concentration (ppm) and volume (L) of the gas sample, respectively, ρ is density (g/mL) and *Vs* is the volume (μL) of the liquid sample, *T* is the temperature (Kelvin) and *M* is the molecular weight of analyte (g/mol). Gas stored bottles were sealed to prevent leaking, then stored at room temperature to be evaporated, and injected into the gas chamber with a syringe during detection.

### 2.4. Planar Optical Waveguide Gas Detection System

**Figure 2:**
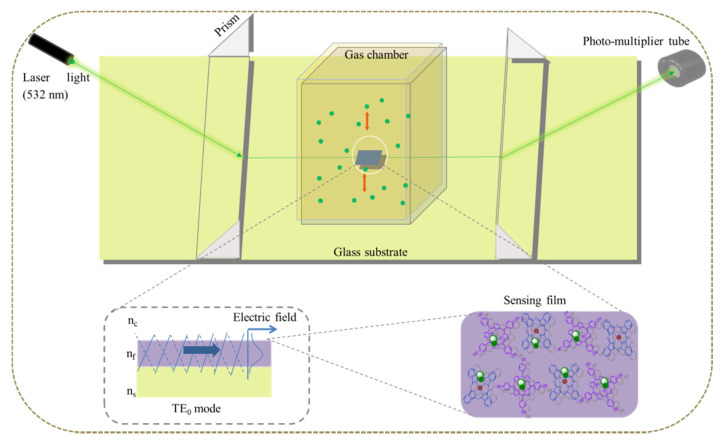
Schematic diagram of the homemade planar optical waveguide (POWG) detection system.

Gas detection was performed in a homemade planar optical waveguide platform (Figure 2) coupled with a semiconductor laser tube with wavelength as 532 nm, beamwidth as 1 mm, and light power as 10 mW (Kochi Toyonaka Co., Ltd., Osaka, Japan); semi-angle of beam incident is set up to 70° during detections, and the laser polarization ratio is 1:1.

Sensitivity in analyte concentration detection is due to evanescent wave, which is part of the guiding light coming out the sensing film surface into the surrounding air. A guiding wave in TE_0_ mode (transverse electric mode) [27] is formed in the sensing film by differentiating the refractive index. This is obtained applying a potassium-ion-exchanged glass substrate with a layer (1–2 μm) of higher refractive index (n = 1.52) with respect to the whole body of the substrate (n = 1.51), and by adjusting manually the propagation angle to 70° in POWG platform [28]. 

The refractive index of air as cladding layer is 1.00, that of potassium ion-exchanged glass substrate is 1.52 [13], and THPP and ZnPc refractive indexes are 1.75, 1.76, respectively [29,30]. A pair of triangular glass prisms (n = 1.78), with a 15 mm distance, was used to introduce the laser light into the sensing layer. To minimize the propagation loss, drops of diiodomethane (refractive index 1.74) were used between prisms and substrate filling the air gap. Refractive indexes of all related materials and gases are listed in Table 1. The sensing film was covered with a 1.3 cm^3^ sealed glass cube as a gas chamber with a gas inlet and outlet tubes. Dry air (purity of 99%, Air Gas Inc., Riverside, China) was used as the carrier gas. The temperature and relative humidity of the environment was controlled with an air conditioning dehumidifier (Haier, KY-26/AB, Haier Group, Qingdao, China). Field Point analog input and output modules (National Instruments, Austin, TX, USA) were used to control and monitor the voltage of the circuit, and the bias potential was kept as 110 V across the whole sensing performance. In all the experiments, the film was first exposed to dry air to achieve a steady baseline, successively was exposed to the analyte gas with a proper concentration; the restoration of the initial state was made by TMA vapor exposure, and then back to air until a stable baseline was achieved. This process refers to one cycle.

## 3. Results and Discussion

### 3.1. Film Characterization

**Figure 3:**
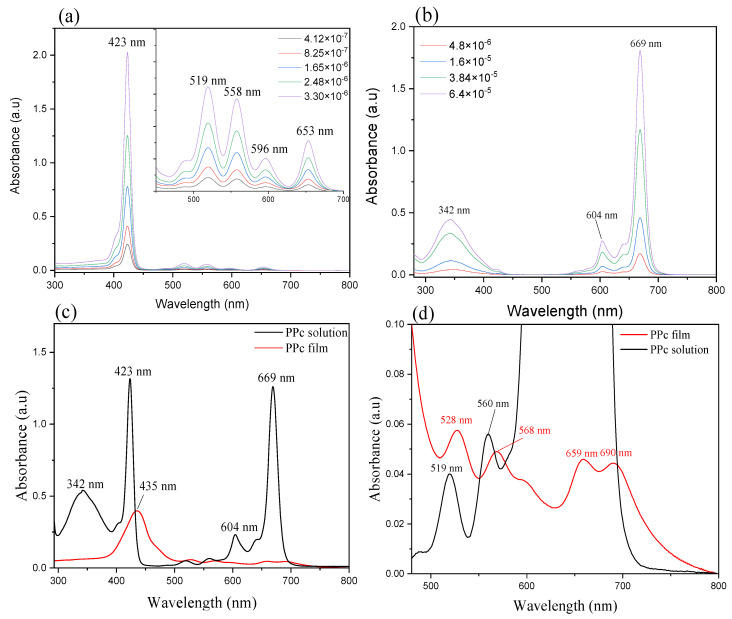
Absorption spectra of (**a**) THPP in DMF solution (4.1 × 10^−7^ mol L^−1^ to 3.3 × 10^−6^ mol L^−1^), (**b**) ZnPc in DMF solution (4.8 × 10^−6^ mol L^−1^ to 6.4 × 10^−5^ mol L^−1^), (**c**) PPc blend before and after film formation, (**d**) magnified spectra of the PPc blend in the range 500–800 nm.

UV-Vis absorption spectra can be used as a powerful tool to determine the chemical and optical properties of these dyes in solution state or film state since both porphyrin and phthalocyanine exhibit strong characteristic peaks. Electronic transitions in these molecules are explained by Gouterman’s four-orbital model [31]. As reported in Figure 3a,b, THPP shows a typical Soret band at 423 nm, and low intense Q bands at 519, 558, 596, and 653 nm and, on the other hand, ZnPc shows a small Soret band at wavelength as 342 nm and high intense Q-bands at 604 and 669 nm; both the macromolecules spectra were recorded in DMF, as solvent.

The molar extinction coefficients (Ꜫ) of THPP and ZnPc in DMF were calculated according to Lambert-Beer law within a concentration range of 1.6 × 10^−6^ mol L^−1^ to 6.4 × 10^−5^ mol L^−1^, and reported in Table 2. The molar extinction coefficient of porphyrin is as high as 6.013 × 10^5^ M^−1^ cm^−1^ at 423 nm, and as low as 9.169 × 10^4^ M^−1^ cm^−1^ at 653 nm; while for phthalocyanine, it is 2.766 × 10^5^ M^−1^ cm^−1^ in the visible spectral region (669 nm), and 6.978 × 10^4^ M^−1^ cm^−1^ at 343 nm. Thus, combining these two molecules may strengthen the absorption sensitivity across the visible region, which is preferred in laser light wave-guiding in our homemade POWG system.

Optical waveguide sensing film requires a nonlinear optical material as a solid film, that can be prepared with preferable optical quality. The non-linearity in POWG was investigated by UV-Vis spectroscopy; the region of interest for optical limiting is in the visible region, where a rather strong change takes place in absorbance intensity before and after gas exposure. In this spectral region, the laser light wavelength should be selected.

The blend of the THPP porphyrin and ZnPc is recorded as PPc in this work. As can be seen in the absorption spectrum in Figure 3c, in the solution mixture, both THPP and ZnPc maintain their original characteristic bands of THPP monomer (423 nm, 519 nm, 558 nm, 596 nm, 653 nm) and ZnPc monomer (342 nm, 604 nm, 669 nm); in Q region the two bands of THPP monomer (596 nm, 653 nm) are overlapped with the intense bands of ZnPc. 

In the film state, reported in Figure 3c,d, the Soret band at 342 nm, which is due to π-π * electron transition on ZnPc macrocycle, is quenched due to the formation of higher degrees of aggregates [32]. The characteristic band of the THPP monomer at 423 nm is broadened and shifted to 435 nm (Δλ = 12 nm). The Q bands change in intensity and are red-shifted from 519 and 560 to 528 and 568 nm; in the film state, the other two Q-bands assigned to THPP porphyrin molecule at 600 and 659 nm, can be observed.

The strong Q band of ZnPc, relative to the electronic transition from the highest occupied molecular orbital (HOMO) to the lowest unoccupied molecular orbital (LUMO), is red-shifted and strongly decreased in intensity. The red shift to 690 nm (Δλ = 21 nm), compared to the monomeric state in solution (669 nm), indicates the formation of J-aggregates [33].

Absorption spectra of the solution and blend film PPc, reveals that there are not metal-to-ligand or ligand-to-metal transitions, because of the filled electronic configuration of zinc(II) ion. THPP and ZnPc molecules could form a composite with zinc(II) ion situated in the center between two porphyrin molecules as an H-type aggregate, which was assured based on the absorption shift. In addition, phenol groups of THPP, can interact through H-bonds with the nitrogen atom of the isoindole linker of ZnPc and is held between the two ZnPc molecules through van der Waals interactions [34]. 

**Figure 4:**
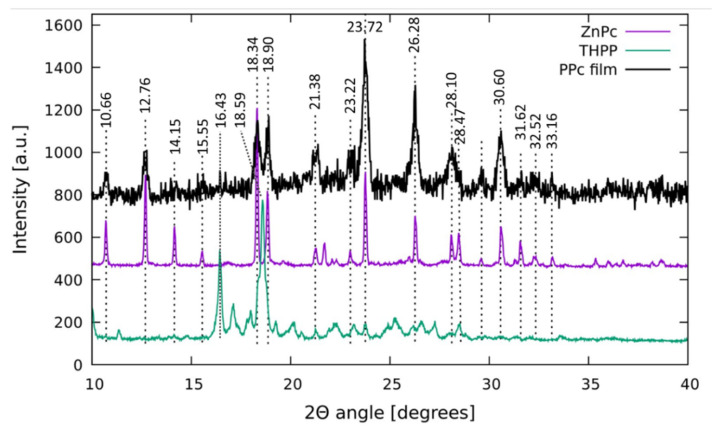
XRD patterns of the blend deposited on the glass substrate (PPc film), THPP powder, and ZnPc powder.

In Figure 4, the XRD patterns of the amorphous phase of the PPc thin film deposited on potassium ion-exchanged glass substrate, compared with those of THPP and ZnPc, are reported. PPc pattern is characterized by strong and broadening peaks at 18.34°, 18.90°, 23.72°, 26.28°, and 30.60° assigned to small crystallites [35]; the predominant peak at 23.72° and other weak peaks indicate that PPc macrocycles preferentially lie parallel to the glass surface with their stacking axes inclining to the substrate [36]. In the film state, ZnPc molecules self-assembled via intermolecular π-π interactions and remains as metastable α form (PDF#21-1986) [37,38]. The ZnPc governs the orientation of the aggregates on the glass, confirmed by the similarity of the PPc pattern, very similar to that of ZnPc with only a small influence of THPP signal at 18.59°.

**Figure 5:**
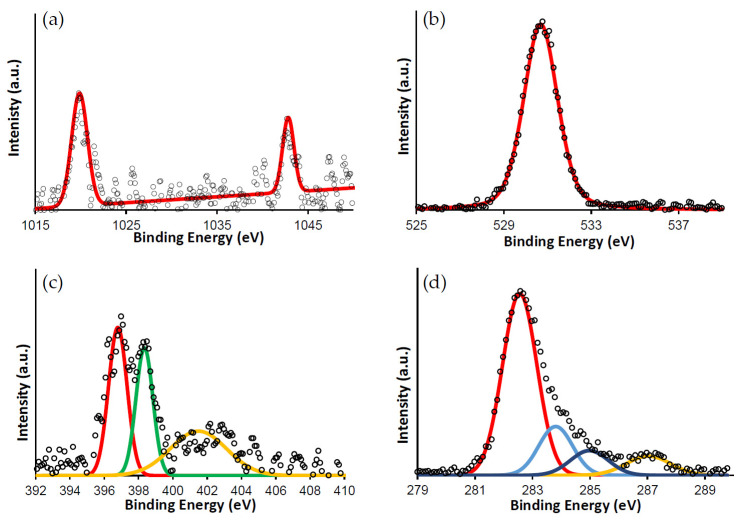
XPS spectra of the PPc blend deposited on glass substrate (**a**) Zn-2p, (**b**) O-1s, (**c**) N-1s, (**d**) C-1s.

In Figure 5 are reported the XPS spectra; as shown in Figure 5a, the peak at 1020 and 1043 eV corresponds to the Zn-2p3/2 and Zn-2p1/2 level, respectively [39] suggesting the bivalent state of zinc ion. O1s spectrum (Figure 5b) shows only one symmetric peak at 1295 eV, indicating the presence of only species as phenolic oxygen rather than multi-component [40]. N1s XPS spectrum (Figure 5c) shows three contributes at 397, 398.5, and 401.2 eV attributed to -N=C- bond, -N-Zn bond, and to pyrrolic nitrogen -NH- of porphyrin and phthalocyanine molecules, respectively [41]. The carbon C1s XPS signals (Figure 5d) show four main peaks at 282.58, 283.8, 284.9, and 287.18 eV assigned to C=C, C-C, C-N, and to C-O bonds, respectively [37]. C1s and N1s spectra display slightly broadened and shift towards lower binding energy, compared to reported literature (401 eV), that can be ascribed to the interaction between film molecules and substrate surface [41,42]. 

### 3.2. Optical Waveguide Analysis

Gas detection with an optical waveguide system was performed using a self-assembled detection system, as described in Section 3.1. The response of the detection system was recorded as output light intensity versus time, following the Equation: $\Delta I=I_{gas}-I_{air}$ and $I=I_{0}\left(1-\alpha Nd_{e}\right)$, where *I_air_*, *I_gas_* represents the output light intensity when dry air and analyte gas is the surrounding gas. *I*, *I*_0_ represent the output and input light intensity, *α* stands for the light absorption coefficient of the film, *N* stands for the number of light refraction within one unit light propagation path, and *d_e_* is the practical propagation depth of the light within sensing film. 

**Figure 6:**
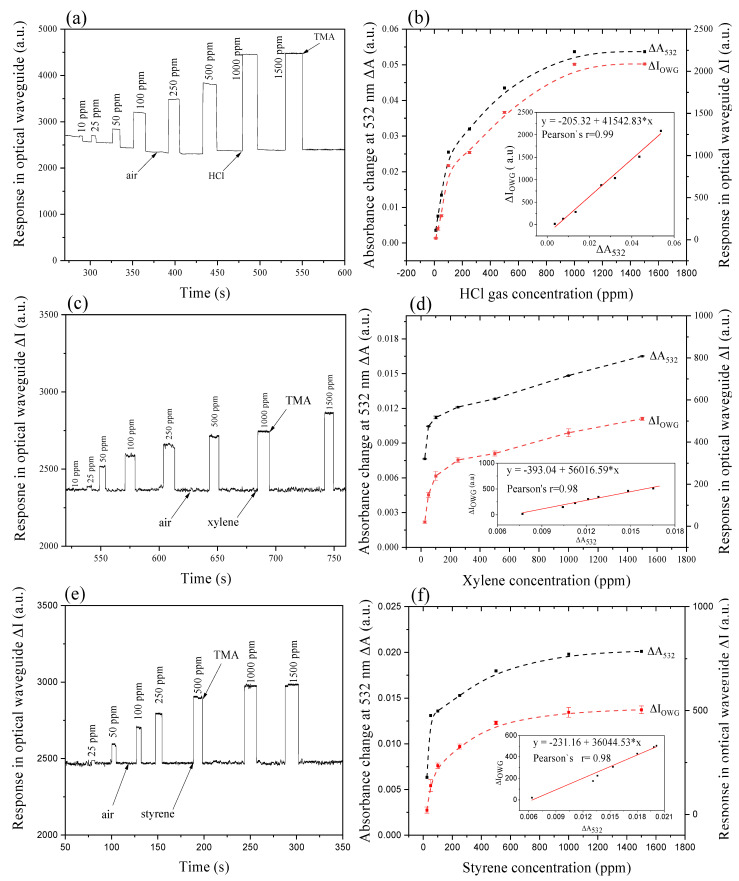
Planar optical waveguide response results along with time (**a**,**c**,**e**) HCl, m-xylene, and styrene exposures (10–1500 ppm; (**b**,**d**,**f**) correlation of the planar optical waveguide response and absorbance change at 532 nm as a function of gas concentration.

UV-Vis spectroscopy was used to select the wavelength of the light source according to the absorbance change at a specific wavelength and to detect the molecular state in film during film-gas interaction in the optical waveguide detection process. A semiconductor laser light tube, with a wavelength of 532 nm, was chosen to act as the guiding light to magnify and transform the chemical information into a readable signal according to the absorbance spectrum analysis. With a guiding light, in the interface of the film and potassium ion-exchanged glass substrate, gas exposure with different concentrations displays signals with distinguished intensity. Response curves along with time are given in Figure 6.

Results show that the self-assembled system can be used in the detection of HCl gas within concentration range 10–1000 ppm (Pearson’s r = 0.99) as shown in Figure 6a,b, m-xylene (Figure 6c,d) and styrene (Figure 6e,f) vapors in the range of 25–1000 ppm (Pearson’s r = 0.99). Refractive indexes of the gas analyte that surround the film surface, are proportional with the evanescent loss of the guiding light, according to the relationship between the attenuated light energy and refractive index [25]. The refractive index of air is equal to 1.0003 at ambient temperature, and that of HCl, m-xylene and styrene are 1.0004, 1.4972, and 1.5469 respectively. Thus, evanescent wave loss is the greatest for styrene and the lowest for HCl gas. However, considering their molecular size (m-xylene = 0.680 nm, styrene = 0.610), the carbon-carbon double bond on styrene molecule, and the hindrance of the methyl group, styrene diffuses more easily and interacts strongly with benzoic π electrons and ethylic π electrons.

In terms of the diffusion ability into the film, HCl has high diffusivity relative to m-xylene and styrene thanks to its smallest molecular size and lightest molecular weight. These response behaviors of the three tested gases are in coincidence with absorbance spectral behavior in terms of the gas concentration range (10–1500 ppm). Response in absorption spectrum and optical waveguide is well correlated, as shown in Figure 6b,d,f (Pearson’s r = 0.9965, 0.9807, 0.9824). 

**Figure 7:**
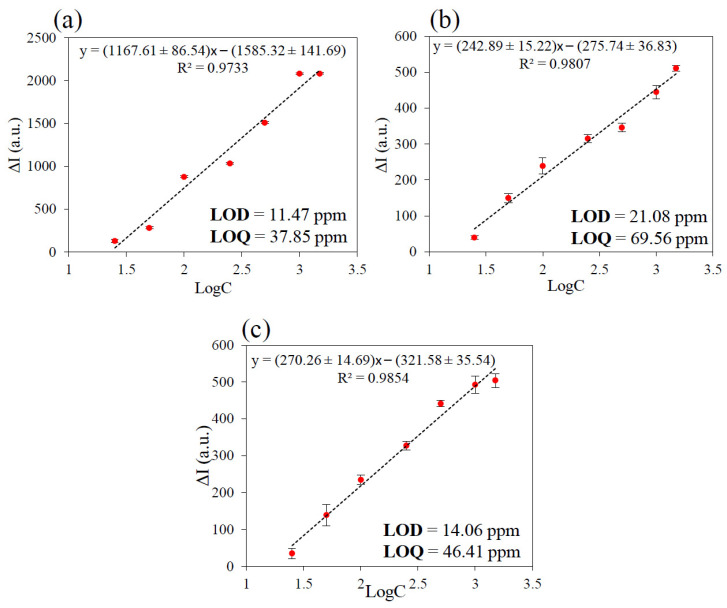
Calibration curve of planar optical waveguide response results for (**a**) HCl, (**b**) m-xylene, and (**c**) styrene from 25 to 1500 ppm.

The response of the POWG system in presence of gases can be linearized as a function of the logarithm of gas concentration as reported in Figure 7. From the calibration curve for all gases under consideration, the LOD and LOQ values are calculated and reported in Figure 7. LOD values for HCl, m-xylene and styrene are 11.47 ppm, 21.08 ppm, and 11.06 ppm, respectively. The LOD results are not particularly low, but they are anyway interesting, in consideration of the wide range of concentration explored. 

Based on the interesting observation in Figure 6a,c,e, films after exposed to HCl, m-xylene, and styrene gases, were re-exposed to TMA saturated gas in POWG, and recovered to the initial state after each gas exposures. Therefore, TMA gas exposure is an effective way to recover the film back to its initial state. 

### 3.3. Spectroscopic Analysis with Gas Exposure

**Figure 8:**
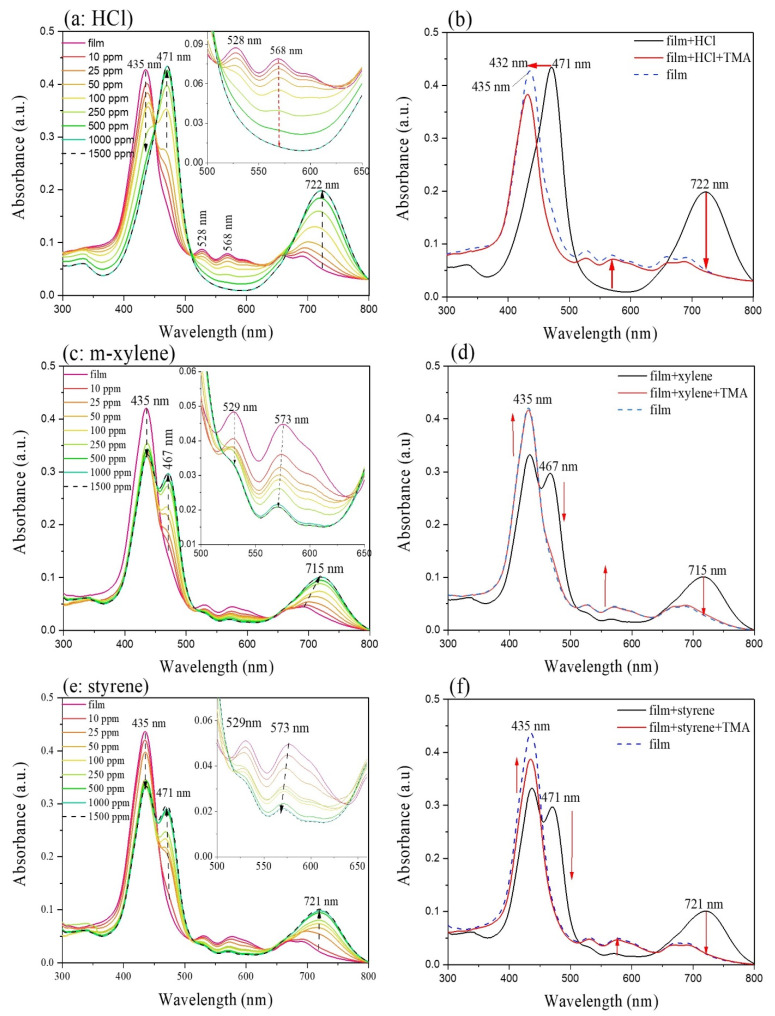
Absorption spectrum of PPc film (**a**) exposing with HCl vapor (10 ppm to 1500 ppm); (**b**) HCl-exposed-film recovery with trimethylamine (TMA) exposure; (**c**,**e**) exposing with m-xylene and styrene vapor (10 ppm to 1500 ppm); (**d**,**f**) m-xylene, styrene exposed-film recovery with trimethylamine (TMA) exposure.

Film-gas interaction, displayed with UV-Vis spectrophotometer at room temperature, provides useful information about the molecular structure transformation, considering remarkable spectral shift and spectral shape. As shown in Figure 8a, the exposure of the prepared film to HCl vapors leads to a red shift of the porphyrin band from 435 nm to 471 nm (Δλ = 36 nm), Q bands at 500–650 nm vanish gradually within 10–1000 ppm gas exposure, and replaced by a strong band at 722 nm with a color-change of the film from yellowish to deep yellow (1000 ppm). 

**Figure 9:**
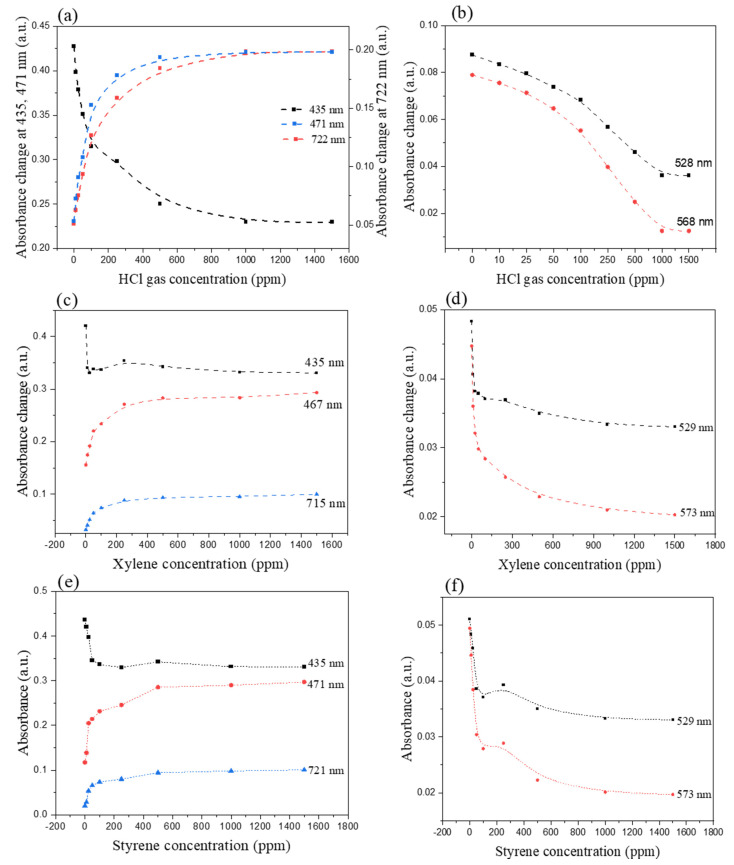
Linearity between absorbance change along with HCl gas concentration in UV-Vis absorption spectrum of PPc film interacting with (**a**,**b**) HCl vapor (10 ppm to 1500 ppm); (**c**,**d**) m-xylene vapor (10 ppm to 1500 ppm); (**e**,**f**) styrene vapor (10 ppm to 1500 ppm).

The absorbance trend, as a function of gas concentrations of HCl, is reported in Figure 9a,b, which shows the absorbance trend of characteristic bands at 471 and 722 nm, and 435 nm in the concentration range 10–1500 ppm. The bands at 528 and 568 nm decreases gradually and disappear at higher concentrations of HCl. All of these bands exhibit no other change when the gas concentration is over 1000 ppm, due to saturation of the blend film. 

THPP has six hydrogen bond donors and six acceptors, and ZnPc owns eight hydrogen bond acceptors without any H-bond-donor. Thus, the exposure to HCl molecules can protonate the porphyrin which can be confirmed by the typical characteristic red-shifted bands at 471 nm and 722 nm and the disappearance of the other Q bands [43,44] due to the formation of aggregates of protonated molecules on the surface. The protonation of porphyrin leads to a distortion of the porphyrin ring with tilting of the pyrrole rings, and also to the rotation of the phenol-ring present in the meso-positions [45]. The de-metalation of ZnPc complex could be possible at a high concentration of HCl, but no remarkable spectral evidence isobserved since the large Q-band at 722 nm overlapped the Q-bands region of phthalocyanine. 

The optimum region for the optical limiting of the film is shown as magnified in Figure 8a. The absorption in this region is due to a high vibrancy level of the first excited triplet state absorption cross-section. The protonated form can be successfully recovered back to the initial state with TMA vapor exposures, as shown in Figure 8b. Based on these results, a green laser light with wavelength 532 nm and TMA vapor was chosen to serve as the light source and recovering gas for the waveguide system.

Exposure of the as-prepared thin film to m-xylene and styrene led to a similar color change observed after HCl exposure, from yellowish to deep yellow. When the film was exposed to various concentrations of analyte gases (10–1500 ppm), the Soret band at 435 nm decreases with the formation of a new band at 467 nm (m-xylene, Figure 8c) and 471 nm (styrene, Figure 8e). Besides, the double bands at around 690 nm are replaced by a peak at 715 nm from xylene and 721 nm for styrene, which increases with the exposed gas concentrations. 

The linearity and absorbance changes trend of the bands along the exposed gas concentration is reported in Figure 9. The Soret band of the blend film at 435 nm is extremely sensitive and drastically decreases already with 10 ppm of m-xylene gas and remains practically constant with the higher concentration (Figure 9c). On the other hand, the new red-shifted band at 467 nm and the Q-band at 715 nm, increase linearly in terms of m-xylene within concentration range 10–50 ppm, while increase gradually within the higher concentrations (50–1500 ppm); no additional spectral changes are observed for both bands with m-xylene concentration over 500 ppm (Figure 9c). The absorbance of Q-bands at 529 and 573 nm decrease linearly within the concentration range (10–50 ppm) while remain constant with concentrations higher than 1000 ppm of m-xylene gas (Figure 9d). 

In the case of styrene gas (Figure 9e), the Soret band of the film at 435 nm decreases linearly with the lower concentrations (0–50 ppm), while remains constant with a concentration of styrene greater than 250 ppm. On the other hand, the red-shifted band at 471 nm increases linearly with the lower concentrations of the gas, and no absorbance change can be observed over 500 ppm of styrene; a similar trend is possible to observe for the Q-band at 721 nm (Figure 9e). Absorbance of the bands at 529 nm and 573 nm linearly decrease in the concentration range 10–100 ppm, while gradually decrease and remain constant with a concentration of styrene higher than 1000 ppm (Figure 9f). 

The blend film for both m-xylene and styrene can be successfully recovered back to the initial state, with TMA vapor exposures, as shown in Figure 8d,f.

Styrene and m-xylene are two planar aromatic molecules and the interaction with THPP and ZnPc macrocycles, which are two π-electrons reach molecules, could be possible through π interaction [46]. In this case, the spectrochemical changes after the exposure of the film to the aromatic gases lead to be similar to the HCl effect. 

Due to their strong molecular polarizability, m-xylene and styrene vapor molecules form supramolecules with PPc molecules by π-π interaction, with a more stable state. The remarkable difference of the spectrum of HCl gas exposure and planar aromatic solvent vapors exposire is the Q bands at 528–568 nm disappeared with HCl exposure while, for the aromatic gases, these two bands decrease gradually even when exposed to highly concentrated (1500 ppm) vapors. Probably, the π-π interaction between these two-guest planar aromatic compounds and the macrocycles in the film, disturb the electron clouds on the macrocycle, leading to the formation of a new state of the macromolecules stacking with small planar molecules. 

As it is possible to observe in Figure 8b,d,f, TMA vapors can restore the initial state of the blend film; in this case, the TMA molecules can deprotonate the previously protonated macrocycles in terms of HCl exposure; the π-π stacking of macrocycles with aromatic solvent vapors was probably disturbed by TMA molecules.

In summary, as shown in Table 3, the extent of maximum band shift (Δλ) is distinguished with 36 nm, 28 nm, and 34 nm for HCl, m-xylene, and styrene vapors, respectively; also, the newly formed band appears at the longest wavelength for HCl (722 nm), and the shortest for m-xylene (715 nm). The absorbance changes at 532 nm, which is chosen as the wavelength of guiding light source in the optical waveguide. From this consideration, the LOD, previously calculated (Figure 7a–c) could be lower by working with different lasers with wavelength, where is observed the major changes in relative absorbance as example 435, 470, and 720 nm (see Figure 8a,c,e).

The spectral changes of the film after m-xylene and styrene exposure are similar to HCl gas exposure with slight differences, and there is a small deviation of xylene and styrene exposed films. The carbon-carbon double bond on styrene molecule leads to stronger π-π interactions with host macrocycles; the two methyl groups on the m-xylene molecule can attenuate the π-π interactions with host molecules, resulting in the weakest change both in Soret band shift and Q band absorbance among the three tested gases. 

The formation of PPc supramolecules with highly conjugated macrocycles and aromatic vapors by π-π stacking leads to attenuation of the energy gap between HOMO and LUMO orbitals, which is suggested by the appearance of the new Q band at longer wavelengths in the UV-Vis spectrum. In this case, the electron transfer from aromatic vapor molecules to PPc macrocycles contributes to the electron cloud on PPc materials due to the molecular polarizability of the guest molecules.

### 3.4. Morphological Analysis

**Figure 10:**
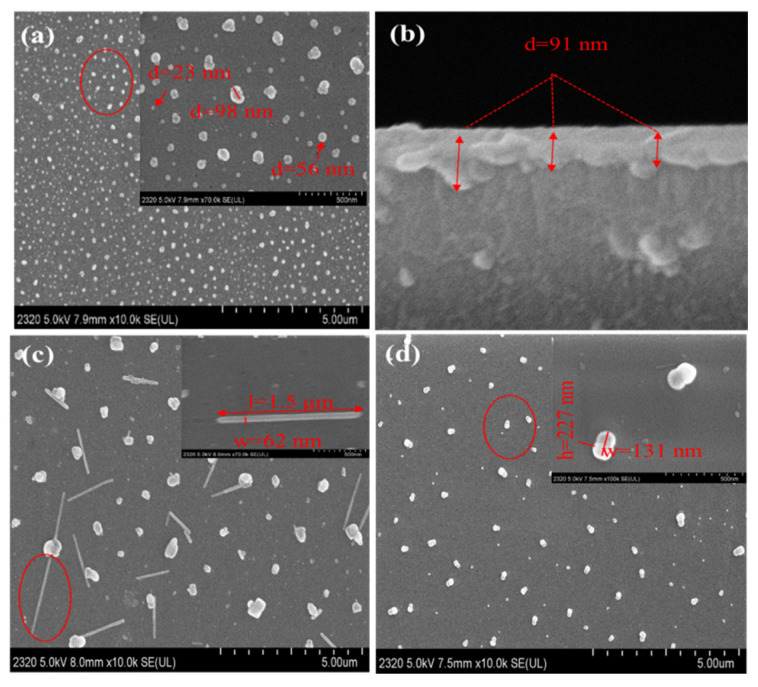
SEM images of PPc film. Before gas exposure: (**a**) surface view and (**b**) cross-section view. After gas exposure: (**c**) HCl and (**d**) styrene.

The blend film of PPc was morphologically analyzed by acquiring SEM images before and after gas exposure. The as-prepared film exhibits uniformly distributed nanoparticles on the substrate with various diameters of 10–100 nm, as shown in Figure 10a. The average film thickness, which was measured in terms of the cross-section of the film deposited on potassium ion-exchanged glass substrate, is around 91 nm with a rather smooth surface, as shown in Figure 10b. The macromolecules tend to distribute uniformly as ball-shaped aggregates via non-covalent interactions on the surface of the potassium ion-exchanged glass substrate. 

After the HCl exposure (Figure 10c), the particles increase in size and sticks of 1.5 μm length (l) and 62 nm width (w) are formed; that is probably due to the aggregation of the protonated PPc molecules, as can be observed from the UV-Vis spectral change. With styrene gas exposure, the regular balls grow larger and are arranged as gourd-shapes with height (h) as 227 nm and width (w) as 131 nm, as can be seen in Figure 10d. This is probably due to swelling of the aggregated films by π-π stacking with small aromatic molecules. The morphological measurement after m-xylene exposure is not performed in this work, since the molecular structure, spectroscopic and OWG response behavior are very similar with styrene. Morphological change is also the reason for the absorbance change in the UV-Vis spectrum and the output light intensity change in the optical waveguide.

## 4. Conclusions

Opto-chemical sensing behavior of metal-free porphyrin and zinc phthalocyanine thin film, with the exposure of HCl, m-xylene, and styrene gases in planar optical waveguide system, was studied. The PWOG response behavior of the film presents sufficient linear correlation with the spectral absorbance changes; the LOD and LOQ values are calculated from the calibration curve, within the range 25–1500 ppm, that is linearized by using the logarithm of the concentration of the gases. Film-gas interaction mechanism is discussed in terms of protonation, π-π stacking between macrocyclic molecules in the sensing film and the analyte aromatic molecules.

This system will be studied in the future with real samples in the presence of multiple components, to improve the application of this device in analysis.

## Figures and Tables

**Table 1:** Refractive indexes (589.3 nm) of related analyte chemicals (101,325 Pa, 298 K).

Chemicals	Refractive Index	Chemicals	Refractive Index
THPP	1.75	Air	1.00
ZnPc	1.76	HCl gas	1.00
Potassium ion-exchanged substrate	1.52	m-xylene	1.50
Glass prism	1.78	Styrene	1.55
Matching liquid	1.74		

**Table 2:** Molar extinction coefficient at different wavelengths of THPP and ZnPc in DMF solution within concentration range 1.6 × 10^−6^ mol·cm^−1^ to 6.4 × 10^−5^ mol·cm ^−1^.

	THPP		ZnPc	
Maximum Bands	Wavelength (nm)	Molar Ext. Coef. (M^−1^ cm^−1^)	Wavelength (nm)	Molar Ext. Coef. (M^−1^ cm^−1^)
Soret band	423	6.013 × 10^5^	343	6.978 × 10^4^
Q bands	520	1.886 × 10^5^	604	1.154 × 10^4^
	558	1.667 × 10^5^	669	2.766 × 10^4^
	596	5.795 × 10^4^		
	653	9.169 × 10^4^		

**Table 3:** Absorbance spectrum analysis of the PPc film with different gas exposures (1000 ppm).

Exposed Gases	Soret Band (nm)	Δλ (nm)	Q Band (nm)	ΔA at 532 nm (a.u)
HCl	471	36	722	0.0837
m-xylene	467	28	715	0.0170
Styrene	471	34	721	0.0201

## Data Availability

The data that support the findings of this study are available from the corresponding author, upon reasonable demand.
